# Challenges and countermeasures of China’s research on “sports promoting adolescent socialization”–data mining based on Weibo and CNKI

**DOI:** 10.3389/fpsyg.2023.1123712

**Published:** 2023-07-18

**Authors:** Yueyan Jiang, Baole Tao, Tianci Lu, Jun Yan

**Affiliations:** College of Physical Education, Yangzhou University, Yangzhou, Jiangsu, China

**Keywords:** adolescent, socialization, physical education, contradiction between supply and demand, data mining

## Abstract

**Objective:**

Understand the public demand for Adolescent socialization, and compare the hot spots in the researches of “sports promoting Adolescent socialization,” so as to clarify the high-quality development direction of sports promoting Adolescent socialization in the new era.

**Methods:**

Collected the Weibo texts about “Adolescent socialization” from April 13, 2017 to June 1, 2022 through python, and used LDA to understand the relevant hot spots. Used City space to collect CNKI studies related to “sports promote Adolescent socialization,” and used keyword co-occurrence network to understand hot spots.

**Results:**

A total of 77,900 original Weibo texts were captured. The theme model identified five potential themes: “School and Family Education,” “Social Participation,” “Community Education,” “Ideological and Political Education” and “Media.” The semantic network showed that the public concerns of each theme include “Adolescent socialization education content,” “relevant policies and regulations,” “Adolescent socialization environment,” “traditional culture” and “publicity ways and examples”0.52 studies were finally retrieved. The keyword co-occurrence network showed that keywords such as “Adolescent,” “Socialization,” “Sports Games,” “School Sports” and “Family Sports” appeared more frequently and had higher intermediary centrality. Researches focused on Socialization, Internet Addiction, Coupling, School Sports, Sports Socialization Media.

**Conclusion:**

The public’s attention to “Adolescent socialization” has three characteristics: subject linkage, object susceptibility, and media diversity. The researches on “Sports Promoting the socialization of Adolescents” has some problems, such as the imbalance of subject supply and demand, the inconsistent way of object supply and demand, and the different content of media supply and demand. The reason is that there is an information island between people and scholars. Existing research results can not meet the demands of the public.

## 1. Introduction

Marx believed that sociality is an essential attribute of human beings. Adolescence is the peak period of individual physical and mental health development and is a critical period of human socialization (Hay, 1994). China has set a policy goal for adolescent development; specifically, adolescents should adapt to and integrate into society more actively and confidently. The high-quality development of adolescent socialization is one of the most important topics in China’s education. Academic research on adolescent socialization offers detailed and in-depth interpretations of this policy. However, due to different perspectives, the public’s interpretation of the policy is inconsistent with that of scholars. The island effect (or island phenomenon) refers to a lack of material circulation and energy exchange between subsystems within an ecosystem (Xie, 2010).This effect reduces a system’s overall efficiency (Fang, 2019). Social informatics states that there is an information island effect in the social system. Three situations can cause such a phenomenon: (1) social subsystems are not related to each other, (2) social information is not shared across subsystems, and (3) social information is disconnected from actual social situations (Ma, 2022).

The ability of sports to promote adolescent socialization is an important area of research and a significant stage of development in human socialization. It is also important in the fields of sports psychology and sports sociology. Studies have shown that sports can improve adolescents’ self-esteem, well-being, and social capabilities (Bedard et al., 2020; Noonan, 2022; Wilson et al., 2022). Adolescents can develop underlying life skills by participating in sports (Super et al., 2021). Physical exercise can also reduce the pressure of teenagers (Norris et al., 1992). Therefore, sports contribute to “adolescent socialization.” However, due to the existence of the information island effect, whether or not research on the influence of sports participation on adolescent socialization can meet the actual needs of the public remains to be discovered.

In the information age, online social platforms have the advantages of high openness, strong interactions, loose environments, and rapid dissemination. Online communication has gradually become an important way for people to express their attitudes and views (Zhang et al., 2022). Through big data analysis, we can effectively refine the public’s universal behavior and needs that are closely related to cultural, economic, social, and other factors (Ren et al., 2017; Li et al., 2021). Weibo is a social platform, similar to Twitter，that enables users to share and spread information instantly and interactively through multimedia forms such as text, pictures and videos (Xi et al., 2021). As a popular social media platform in China, Weibo provides a large sample of adolescents (Wang and Zhao, 2022). According to the 2020 user development report by Weibo, the number of monthly active users on Weibo reached 511 million. Until to September 2020, the post-1980s, post-1990s, and post-2000s generations account for 96% of the total users on the platform. This population is an important segment of adolescents’ social participation. Compared with traditional questionnaire data, network data more accurately reflect real life because the source of the data (i.e., the platform user) is in a natural state. On weibo, people can freely talk about their real opinions on some topics through the anonymity of the Internet. Even with negative opinions, people on weibo do not have to worry about being blamed from people they know (e.g., parents, friends, etc.). The technology acceptance model(TAM)postulates that the individuals’ intentions are influenced by their attitudes (Camilleri and Kozak, 2022). Thus, the use of weibo for virtual interactions related to adolescent socialization depends entirely on the will of the individuals. On Weibo, users’ attitudes toward adolescent socialization are truly reflected.By examining the Weibo data, the actual needs of the public in terms of the social development of adolescents can be obtained. Therefore, this research used text mining to mine original microblog data on adolescent socialization. We then compared the Weibo data with data from the literature, specifically from articles published on the topic “sports promote adolescent socialization” to explore the future direction of this research.

## 2. Materials and methods

### 2.1. Microblog data capture and preprocessing

Because the term “Adolescent socialization” is professional, people rarely use this phrase directly in the actual network environment. Data capture directly with the keyword “Adolescent socialization” will result in the omission of a large number of relevant data. Therefore, data capture was conducted with the keyword “Adolescent + society” instead. Since the research mainly focused on the focus of the public on “Adolescent socialization” since the promulgation of the Chinese policy “Medium-and Long-Term Adolescent Development Plan (2016–2025),” the original microblog data from April 13, 2017 to June 1, 2022 were captured. A total of 106,413 original microblogs were captured, and 77,900 original microblogs were obtained after using Pandas to delete duplicate text data. Jieba was used for chinese word segmentation and deactivation.

### 2.2. Source and screening of literature data

CNKI was used as the literature source. CNKI is one of the authoritative platforms for the exchange and dissemination of knowledge and information resources in China at present, and the documents it collects can relatively representative reflect the overall picture of the research on “sports promoting Adolescent socialization” in China. In terms of search scope, the definition of “Adolescent socialization” was derived according to the definition of individual socialization, that is, the process of Adolescent’s acceptance of social culture, the whole process of Adolescent’s growing from natural or biological person to social person and gradually adapting to social life (Li, 2009). Because the definition of Adolescent socialization covered a wide range, in combination with the actual publication of relevant research, in order to search as much as possible the relevant research on sports promoting Adolescent socialization, the research retrieval conditions were selected as “theme: (‘sports’ and’ Adolescent socialization’) or theme: (‘sports’ and ‘Adolescent social development’).” The retrieval time was from the establishment of the database to June 1, 2022. 281 articles were retrieved. After excluding non-periodical literature, repetitive literature, irrelevant and non-research literature, 52 results were obtained, and the format was Refworks.

### 2.3. Microblog data analysis

Text data is one of the main sources of network data. Using text mining technology to convert semi-structured text data into structured data for processing and analysis, and apply it to scientific research, has become one of the common means in economics, information science, management science and other fields (Xia, 2022).

Topic model is used to cluster the implicit semantics of the target text set by unsupervised learning, which can help researchers quickly and intuitively mine the internal topic distribution from a large number of texts. Latent Dirichlet Allocation (LDA) topic model is a kind of unsupervised learning algorithm. It does not need to add document labels manually, but only needs to specify the number of topics (K). In order to determine the best K value, PyLDAvis was used to visualize the results of the theme model, and bubble charts were used to display all topics. Each bubble in the bubble chart represented one topic, the size of the bubble represented the importance of the topic in the corpus, and the distance between bubbles represented the similarity between topics. When each topic was relatively independent, the K value was optimal.

Pointwise Mutual Information (PMI) can measure the correlation between two things. Because high-frequency words are easy to mislead computer co-occurrence analysis and processing results, such as “I,” “you” and other words have a high co occurrence frequency, but there is no actual relationship between words. To solve this problem, PMI is commonly used in semantic network analysis.

Analyzing the affective tendencies of texts can be carried out for words, sentences and paragraphs, with the affective analysis of words being the basis for the other two analyse (Xu et al., 2019). We used GooSeeker, an analytical software package based on a textual sentiment dictionary to distinguish which texts had negative, positive and neutral affective tendencies.

### 2.4. Literature data analysis

CiteSpace 6.1 R2 version was selected for data visualization analysis. CiteSpace is a scientific metrology software developed by Professor Chen Chaomei based on Java, which is currently widely used in scientific text mining and visual analysis. The specific settings of the study were as follows: the analysis time was selected to June 2022, the default time zone partition was 1 year, the node type was selected as a keyword, and the threshold value was selected as Top N = 30, that is, the top 30 nodes in each time zone slice were selected. Gephi 0.9.2 software was selected to optimize the visualization results’ pictures.

## 3. Results

### 3.1. Analysis results of original microblog data

#### 3.1.1. Word frequency statistics results

**Figure 1:**
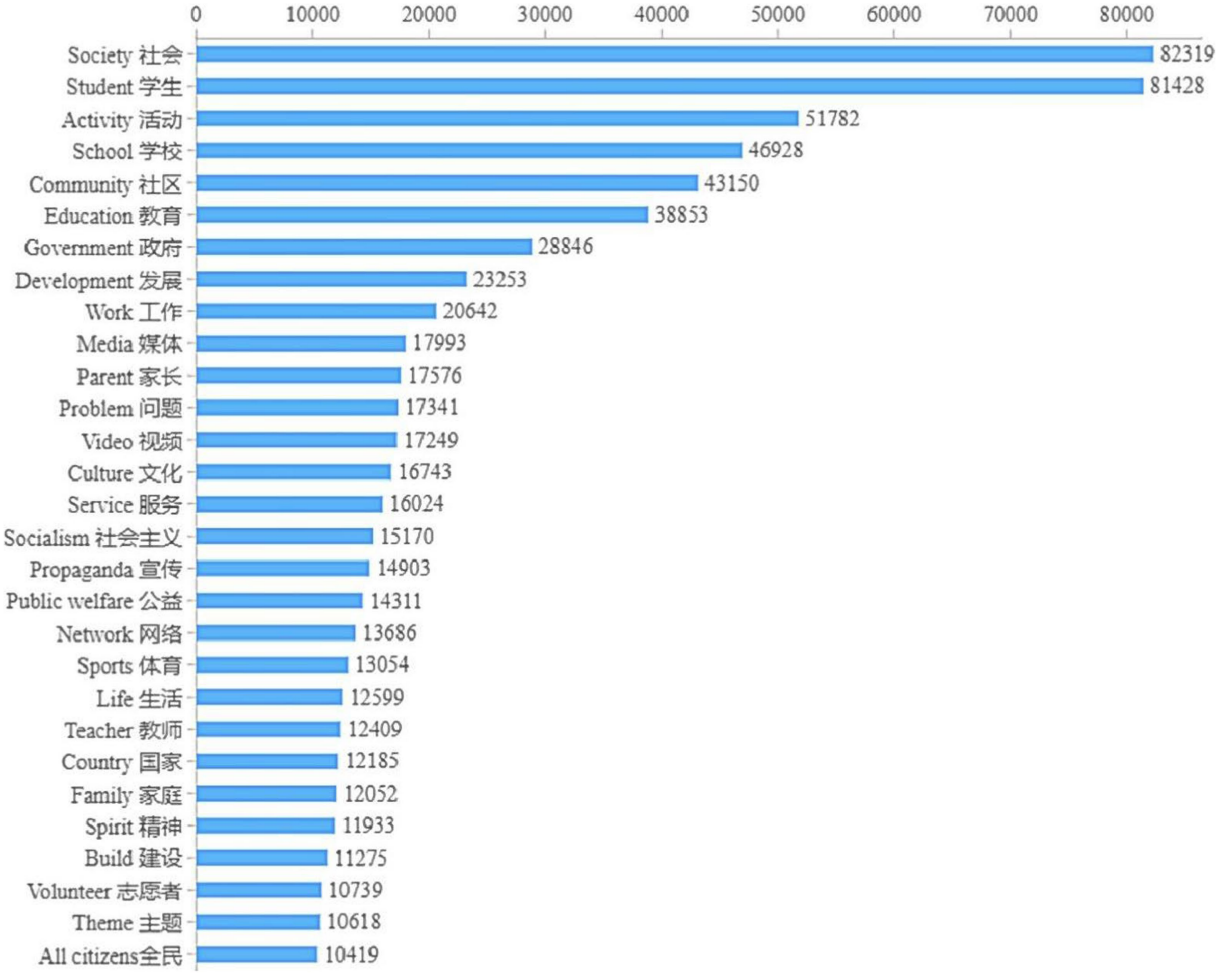
Word frequency statistics.

The word frequency statistical chart (Figure 1) shows that, in addition to phrases closely related to the socialization of adolescent, such as “society” and “students,” words with high public concern are also concentrated in the education, development and work of schools, communities and governments.

#### 3.1.2. Theme analysis results

**Figure 2:**
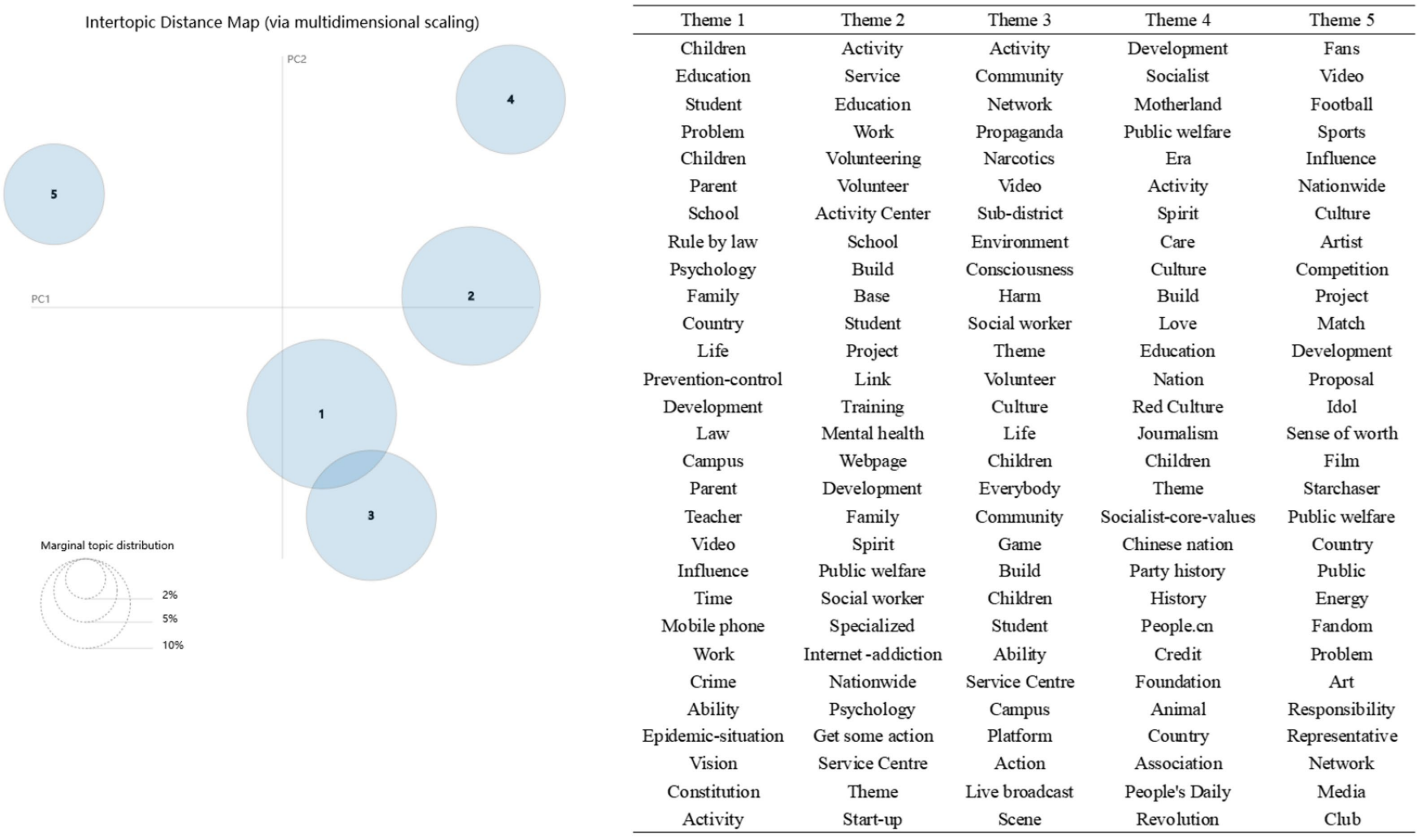
Theme distribution and Top 30 subject words of each theme.

The bubble chart shows that the best K value is 5. The first 30 subject words corresponding to each bubble are shown in Figure 2. The researchers name the subject according to the subject words. Theme one contained such keywords as “Education,” “Children,” “School” and “Family.” Therefore, the topic was named “School and Family Education,” which was the topic most concerned by microblog users. The second theme bubble contained the words “Service,” “Volunteer,” “Base,” “Project” and “Training,” so it was named “Social Participation.” The representative words in the third theme were “Community,” “Sub-district,” etc., so this theme was named “Community Education.” The representative theme words of the fourth theme were “Socialism,” “Motherland,” “Spirit,” “Culture,” etc., so this theme was named “Ideological and Political Education.” The representative theme words of the fifth theme included “Fans,” “Artist,” “Network,” “Media,” etc., so this theme was named “Information Transfer Mode.”

#### 3.1.3. Semantic network analysis

**Figure 3:**
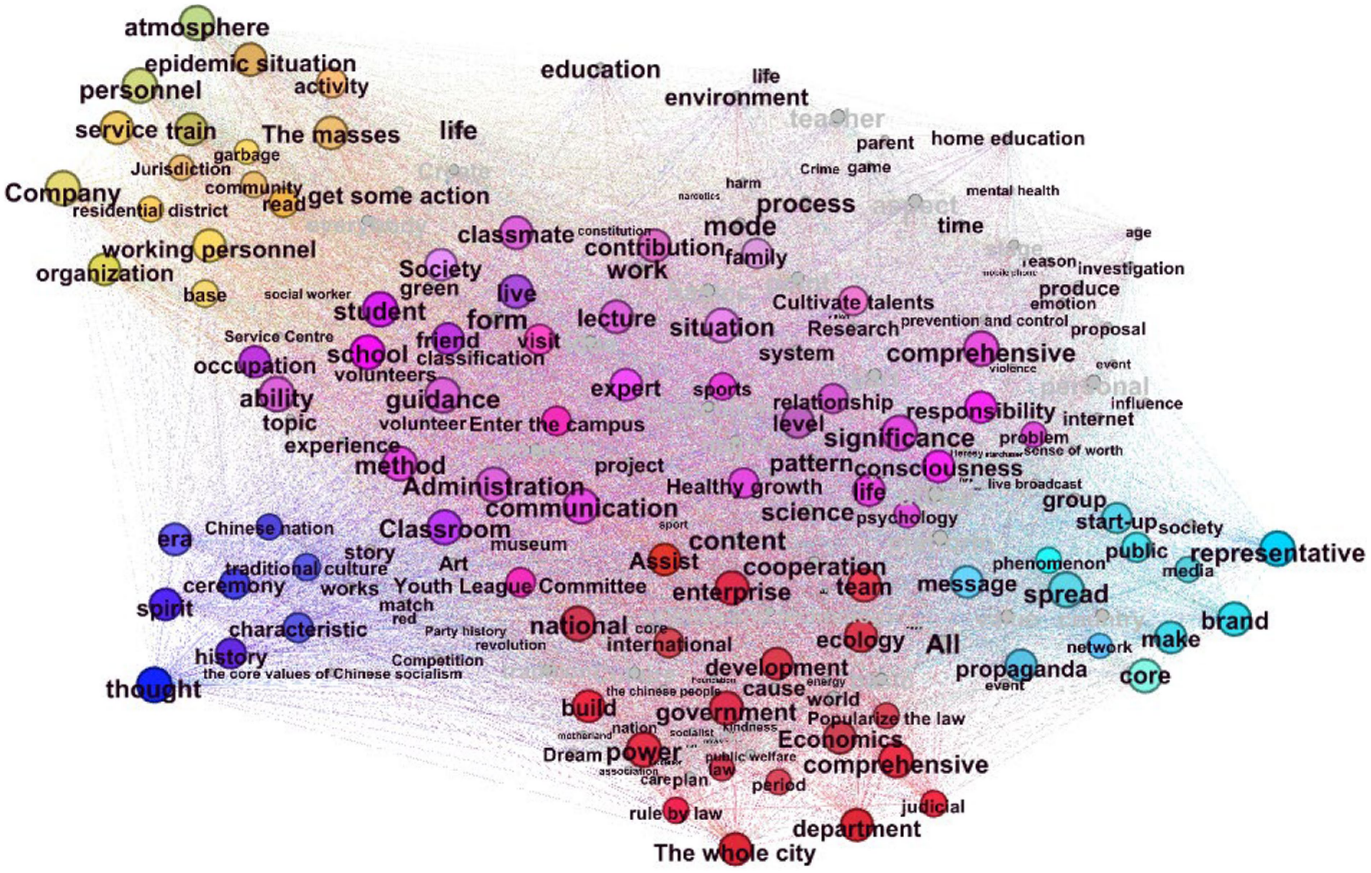
Semantic network analysis.

To further explore the relationship between various themes, the public’s focus on each theme is shown in Figure 3. Theme one “School and Family Education” focuses on the contents, teaching methods and participants of Adolescent socialization education. Theme two “Social participation” focuses on the establishment of relevant policies and regulations of the government and the assistance of third parties such as enterprises. Theme three “Community education” focuses on the construction of a socialized environment for adolescent and related services.Theme four “Ideological and political education” focuses on the inheritance of traditional culture and the promotion of national spirit. Theme five “Media” focuses on publicity channels and model setting, and each theme is closely linked.

#### 3.1.4. Overall affective tendencies

After we analyzed the affective tendencies of the keywords in the collected texts, the results show the public’s attitudes. Among the 77,900 original Weibo texts, 17,794 texts were categorized to have negative sentiments and 56,169 texts were judged to have positive tendencies, accounting for 22.85 and 72.10% of the total number of samples, respectively. In addition, there were 3,937 texts that were not found to have obvious emotional tendencies, accounting for 5.05% of the total number of samples. Among them, the texts with positive emotional tendencies indicated that social media users exhibited positive emotions to “Adolescent socialization” through their texts, including “outstanding,” “full of youthful spirit,” “honest,” “healthy,”et al.

### 3.2. Analysis results of journal literature data

#### 3.2.1. Keyword co-occurrence analysis

**Figure 4:**
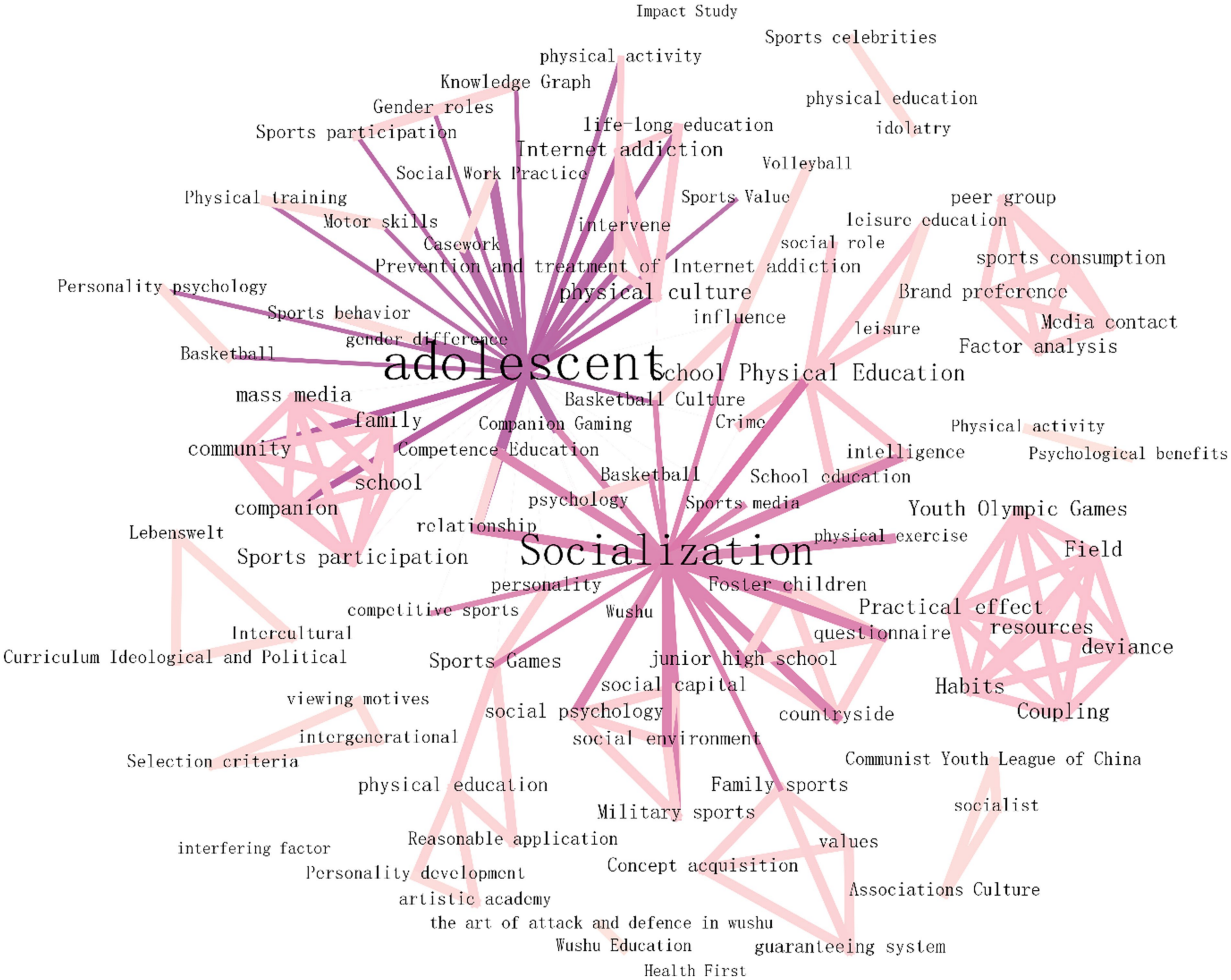
Co-occurrence analysis of key words related to sports promoting Adolescent socialization.

Keyword co-occurrence analysis is to analyze the frequency of keywords provided by the authors of each literature in the collected literature, and grasp the research trends and hot issues in this field by analyzing the frequency of keywords (Li and Chen, 2016). Leave the remaining parameters unchanged, set the node type as the keyword, select Pathfinder, and use Gephi to optimize the map. The resultant keyword co-occurrence map is shown in Figure 4. Keywords in the co-occurrence spectrum, the number of nodes (N) is 94, the number of connections (E) is 185, and the network density (Density) is 0.0423. The top 10 key words in the research frequency of “sports promote Adolescent socialization” are: Adolescent, socialization, school sports, physical exercise, Internet addiction, sports media, competitive sports, sports games, family sports, and social roles. In keyword co-occurrence analysis, the centrality can indicate the importance of a node in the co-occurrence network. The higher the centrality, the greater the impact of the node on the entire research field. Among the keywords in the co-occurrence map, the key words with higher intermediary centrality are: Adolescents (0.56), socialization (0.41), sports games (0.11), school sports (0.08), family sports (0.08). The high-frequency keywords in the research of “sports promoting Adolescent socialization” are basically consistent with the high school prepositional words, which shows that the above keywords have significant influence in the research network of “sports promoting Adolescent socialization.”

#### 3.2.2. Summary of hot topics

**Figure 5:**
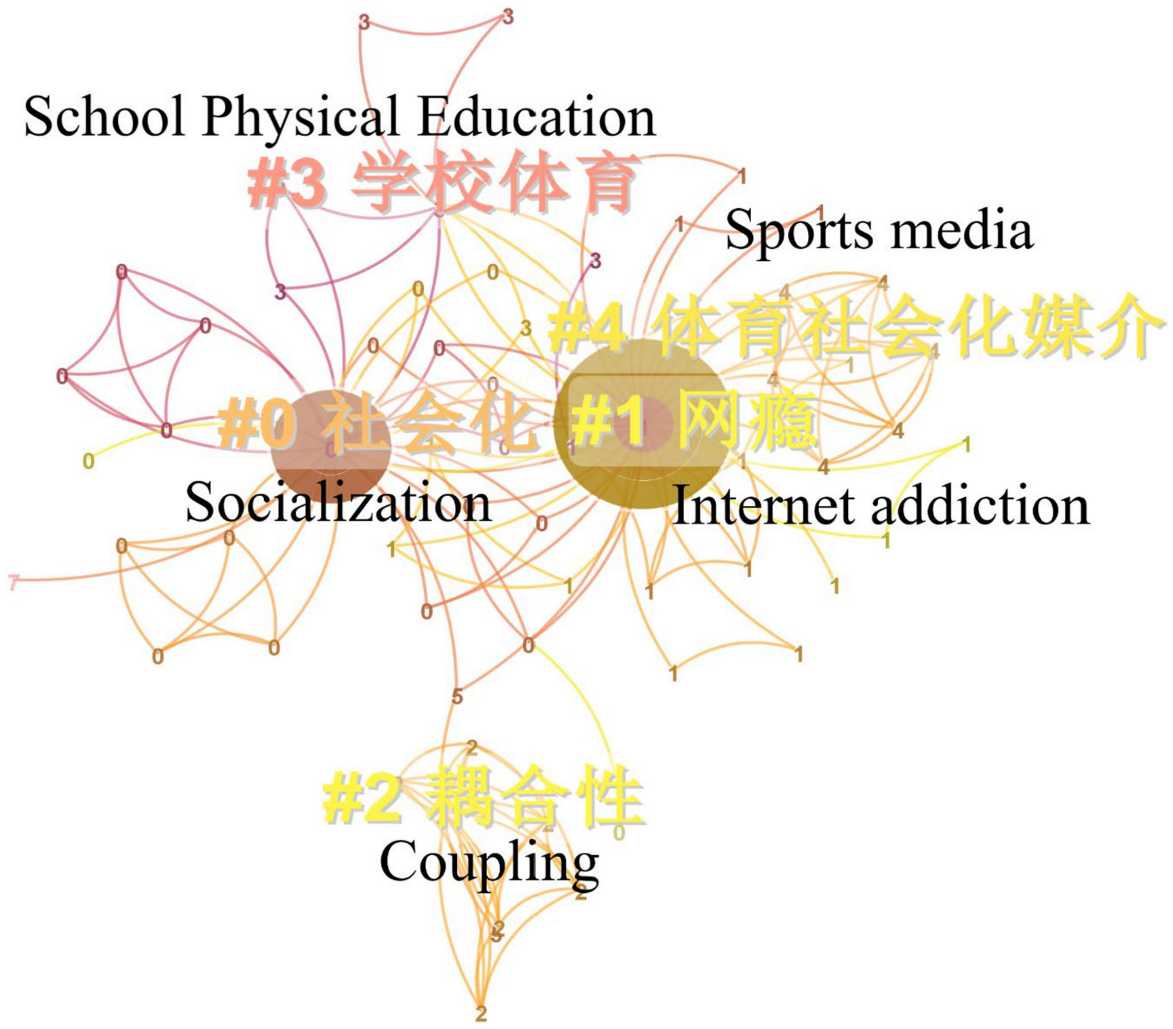
Clustering analysis of keywords related to sports promoting Adolescent socialization.

Set other parameters as above, select Find Clusters, and use Log-Likelihood Ratio to cluster analysis on research keywords related to “sports promote Adolescent socialization.”Cluster analysis results show that the Modularity Q (Q value) is 0.6996, and the Mean Silhouette (S value) is 0.9001 (Figure 5). Q value can reflect the stability of clustering network, and generally greater than 0.3 indicates that the clustering structure is stable. S value can reflect the similarity of nodes within the cluster, and generally greater than 0.5 means that the internal matching degree of the cluster is high (Li and Chen, 2016).

According to the results of keyword clustering analysis, the keywords of the relevant literature on “sports promoting Adolescent socialization” included in CNKI are mainly concentrated in the following five clusters: socialization, Internet addiction, coupling, school sports, sports socialization media. See Table 1 for the representative keywords of each label.

**Table 1:** Keywords clustering and distribution of research on sports promoting Adolescent socialization.

No.	Label	Representative keywords
#0	Socialization	social environment, Basketball Culture, questionnaire, Social attribute of sports
#1	Internet addiction	Internet addiction, adolescent, physical activity, Socialization, life-long education
#2	Coupling	Coupling, Habits, Practical effect, Youth 0lympic Games, deviance
#3	School physical education	School Physical Education, School Education, intelligence, leisure education, leisure
#4	Sports media	Sports socialization media, mass media, School, companion, community

## 4. Discussion

### 4.1. Characteristics of the focus of public concern

On the whole, the public has a positive attitude toward adolescent socialization. The essence of the public’s concern about adolescent socialization remains on adolescent education. Based on the basic elements of education—education subject, education object, and education influence—this public focus can be summarized in the following three aspects.

#### 4.1.1. Cooperation between educational subjects

Thematic analysis shows that people pay close attention to the main educational subjects (schools, families, and communities). Semantic network analysis shows that, while adolescent socialization is closely linked with home and school, the construction of the community environment is also crucial. In January 2022, the Family Education Promotion Law of the People’s Republic of China was implemented, which clearly mentioned that “family education should meet the requirements of close integration and coordination of family education, school education, and social education” (page).The cooperative education of family, school, and community is essentially a macro-educational concept. In the process of adolescent socialization, the family is the most primitive social environment, which is an important factor to establish adolescent social cognition and behavior. School is the continuation of family socialization and plays a more important role in the process of human socialization. Research shows that family-, school-, and community-coordinated education will help adolescents to correctly, comprehensively, and harmoniously socialize, and will lead the development of education into a new era (Li, 2018; Wu, 2018).

Structural functionalism holds that a society is composed of groups, classes, and social settings, and all parts of the social system need to play a role in this coordination to maintain the benign operation of the society. Based on structural functionalism, the results of network semantic analysis show that the public’s concern for the linkage between family, school, and community is mainly focused on the “strong link” of the content and method of adolescent socialization through education. Participation in this type of education usually requires parents to have more time, energy, skills, and knowledge. High-intensity work and uneven educational resources of people in different social strata make them unable to solve the educational problem of adolescent socialization. With this “strong link” between families and schools, people need to acquire the relevant skills of adolescent socialization education to ensure the healthy growth of adolescents and cultivate their professional knowledge of life consciousness, social responsibility, and so on through going to school, attending lectures, parents’ classroom, and other ways. In terms of social settings, people in the new era have put forward higher requirements for the community educational process of adolescent socialization. The community is not only a bridge connecting families and schools, but also needs to coordinate and integrate voluntary cooperation between parents in the community, organize other social education resources, carry out activities that are beneficial to adolescents, and establish a base for adolescent socialization.

#### 4.1.2. Object susceptibility

Adolescents are in a period of psychological transition, and their independent consciousness and self-consciousness are growing. They are eager to be rid of the guardianship of adults (especially their parents). The needs of adolescents in terms of social identity, social integration, and other aspects increase significantly compared with those in childhood, and their participation in peer, school, and social interactions improves. Likewise, they have a greater desire for social integration with the new environment (Wang et al., 2021; Yao and Cheng, 2021). However, the psychological development of adolescents is not yet mature, and their ideas and behavior patterns are vulnerable to external influences. As a popular subculture among adolescents, fandom culture has become an important part of adolescents’ lives, and they can quickly integrate into social interactions with the help of popular subcultures. However, abnormal idolatry and praise of negative idols are not conducive to the healthy and positive development of adolescents. Therefore, people must pay more attention to subcultures in adolescent socialization.

#### 4.1.3. The role of the media

The analysis of the microblog themes and semantic networks shows that, in recent years, under the guidance of the mainstream media, the public has paid significant attention to topics such as “the cultivation of socialist core values,” “the promotion of excellent traditional Chinese culture,” and “the inheritance of the Chinese national spirit” among adolescents. The cultivation of adolescents’ socialist values is one of the most important topics of adolescent socialization in China. The three-level value theory of state, society, and the individual asserts that the transmission of national values occurs through implantation (Xu and Yang, 2022). In 2010, Professor Zheng Hangsheng put forward the “social mutual construction theory,” stating that the individual is the ultimate unit of society, and society is the context of individual existence (Yang and Zheng, 2010). Socialist ideological and political education is a diversified social practice that needs to be examined from various aspects and implemented in a variety of cultural forms. Based on the social mutual construction theory, ideological and political education should not be limited to specific areas, such as individuals or schools, but should be integrated into all kinds of publicity.

### 4.2. Challenges of China’s research on sports promoting adolescent socialization

#### 4.2.1. Lack of research on the linkage of educational subjects

Keyword co-occurrence analysis shows that the relevant research has tended to discuss the role and influence of school sports, family sports, and social sports on adolescents’ socialization. Although some scholars have proposed a multi-sport platform led by school sports and participated in by families, schools, and communities (Li et al., 2021), this is only a theoretical discussion. How school sports, family sports, and social sports interact needs to be examined empirically.

#### 4.2.2. Educational approaches are relatively backward

In April 2022, the “Physical Education and Health Curriculum Standards for Compulsory Education” were released; they specifically mentioned that emerging sports should be offered on campuses, enrich the content of campus sports, and be allowed to mobilize students’ interest in participation. Research shows that sports can promote the establishment of young people’s values. Being constrained by various rules in sports participation can also promote the formation of social behavior norms, which is conducive to the formation of social and cultural values (Chamberlain et al., 2021). The traditional form of school sports organization no longer promotes the socialization of young people. Keywords in the co-occurrence results, such as “multi-player sports,” “community culture,” “sports celebrities,” and “sports games,” indicate that the relevant research on how sports promote youth socialization has begun to pay attention to emerging sports organization forms, but the scope of relevant empirical research is small. At present, some physical education teachers in China still face some challenges, such as insufficient understanding and solidification of teaching ideas, leading them to continue operating under the influence of traditional physical education models.

#### 4.2.3. Inadequate media publicity

The results of the keyword cluster analysis show that the research on sports promoting youth socialization has examined the role of the media in sports socialization, but the focus of relevant research on youth socialization is insufficient. The public has paid much attention to such topics as “the cultivation of socialist core values,” “the promotion of excellent traditional Chinese culture,” and “the inheritance of the Chinese national spirit,” which are also important content in sports education. While encouraging young people to participate in sports, the media should also promote physical education.

The causes of the above problems can be analyzed from two aspects: the contradiction between the advancement of research and the lag of reality; and the contradiction between the importance of sports and the degree of public recognition of this. Under the guidance of national policies, the research on sports promoting youth socialization has been greatly developed in theory. From the popular keywords, we find that relevant studies have explored the impact of sports on youth socialization, ranging from the social attributes of sports to specific sports events. Different from this rich theoretical research, the empirical research on sports and youth socialization is extremely weak. This means that the theoretical research results cannot be transformed into real physical education practices in schools; moreover, the lack of empirical research also means that the theoretical research results cannot be verified effectively. As a result, physical education, in practice, cannot meet the actual needs of the public for youth socialization. Although the essential function of sports education has been highly valued by the state and academia, its important role has not been fully recognized by the public. On the one hand, due to the cultural roots of examination-oriented education in ancient China’s imperial examination system, people still despise sports compared with knowledge. On the other hand, some physical education teachers neglect the role of sports in promoting youth socialization.

### 4.3. Countermeasures of China’s research on sports promoting adolescent socialization

Harold Lasswell, an American scholar, once proposed the 5 W model (Who, Says What, In Which Channel, To Whom, With What Effect), which constructs the theoretical framework of communication. Later scholars added the feedback link so that the model became a complete and sustainable process composed of audiences, feedback information, and communicators (Hu, 2021). Therefore, the 5 W model can be used to solve problems.

#### 4.3.1. Subject (who)

To give full play to the unique role of sports in the socialization of young people, it is necessary to establish a diversified team. We must pay attention to the training of physical education teachers. This involves strengthening the cultivation of ideological and political education and sports education among physical education teachers, and improving the overall quality of their teaching. Physical education and other disciplines complement each other and work together to encourage young people to apply the socialized skills acquired in physical education to the study of other subjects. Therefore, the school cooperates with families, communities, or other social organizations to promote the healthy socialization of young people.

#### 4.3.2. Content (says what)

The “socialization consciousness” of young people is the sensitivity to people, organizations, social interactions, and the judgment of social values. It is a sensitive understanding of the necessity of their own socialization and the accumulation of social capital. Only when adolescents have a certain sense of “socialization consciousness” can they have a high degree of socialization demand, acquire social capital, and pay attention to their own and others’ evaluations, and finally be willing to accept socialization education. In addition, we should cultivate the socialization ability of young people. This refers to the knowledge, skills, and methods related to the law of individual social development, including interpersonal communication, social adaptation, and so on.

#### 4.3.3. Way (in which channel)

The high-quality development of sports in promoting the socialization of young people needs to be examined in relation to national policies; excel in the planning of regional linkages; coordinate resources; build a school, family, and community information-sharing platform; and improve the theoretical transformation and practical feasibility of research.

#### 4.3.4. Object (to whom)

As society pays more attention to the socialization of young people, a trend of gradual generalization emerges. While focusing on young people, research should also explore the education of those who are in close contact with young people, such as parents.

#### 4.3.5. Evaluate (with what effect)

The evaluation of how sports promote youth socialization mainly includes three points. First, object participation is the degree of participation that reflects the influence of education on sports and youth socialization through statistics on the total number and coverage of objects. Second, object satisfaction refers to the degree of satisfaction of the object in relation to the approach and content of sports and youth socialization education through online and offline methods. Finally, the development of the socialization process of young people can be studied through quantitative and qualitative research on adolescents, parents, physical education teachers, and relevant experts. In doing so, the development of adolescents’ socialization processes can be directly or indirectly reflected.

## 5. Conclusion

Based on the research results of microblog and CiteSpace, this study found challenges and countermeasures of China’s Research on “Sports Promoting Adolescent Socialization.” As an important part of education, physical education plays an irreplaceable role in the process of “youth socialization.” The existing research on “sports promoting youth socialization” is still unable to meet the actual needs of the people. To solve this problem, it is necessary to make the research on “sports promote the socialization of young people” develop in a down-to-earth manner while conforming to the theme of the times and the research upsurge.

## Author contributions

YJ, BT, and JY designed the study. YJ, BT, and TL collected, analyzed, and visualized the data. YJ wrote the main manuscript text. JY administrated the study. All authors contributed to the article and approved the submitted version.

## Funding

This work was supported by the National Social Science Fund of China (22ATY007).

## Conflict of interest

The authors declare that the research was conducted in the absence of any commercial or financial relationships that could be construed as a potential conflict of interest.

## Publisher’s note

All claims expressed in this article are solely those of the authors and do not necessarily represent those of their affiliated organizations, or those of the publisher, the editors and the reviewers. Any product that may be evaluated in this article, or claim that may be made by its manufacturer, is not guaranteed or endorsed by the publisher.
